# SBDS Expression and Localization at the Mitotic Spindle in Human Myeloid Progenitors

**DOI:** 10.1371/journal.pone.0007084

**Published:** 2009-09-17

**Authors:** Claudia Orelio, Paul Verkuijlen, Judy Geissler, Timo K. van den Berg, Taco W. Kuijpers

**Affiliations:** 1 Sanquin Research and Landsteiner Laboratory, Department of Blood Cell Research, Amsterdam, The Netherlands; 2 Emma Children's Hospital, University of Amsterdam, Academic Medical Centre (AMC), Amsterdam, The Netherlands; Tel Aviv University, Israel

## Abstract

**Background:**

Shwachman-Diamond Syndrome (SDS) is a hereditary disease caused by mutations in the *SBDS* gene. SDS is clinically characterized by pancreatic insufficiency, skeletal abnormalities and bone marrow dysfunction. The hematologic abnormalities include neutropenia, neutrophil chemotaxis defects, and an increased risk of developing Acute Myeloid Leukemia (AML). Although several studies have suggested that SBDS as a protein plays a role in ribosome processing/maturation, its impact on human neutrophil development and function remains to be clarified.

**Methodology/Principal Findings:**

We observed that SBDS RNA and protein are expressed in the human myeloid leukemia PLB-985 cell line and in human hematopoietic progenitor cells by quantitative RT-PCR and Western blot analysis. SBDS expression is downregulated during neutrophil differentiation. Additionally, we observed that the differentiation and proliferation capacity of SDS-patient bone marrow hematopoietic progenitor cells in a liquid differentiation system was reduced as compared to control cultures. Immunofluorescence analysis showed that SBDS co-localizes with the mitotic spindle and *in vitro* binding studies reveal a direct interaction of SBDS with microtubules. In interphase cells a perinuclear enrichment of SBDS protein which co-localized with the microtubule organizing center (MTOC) was observed. Also, we observed that transiently expressed SDS patient-derived SBDS-K62 or SBDS-C84 mutant proteins could co-localize with the MTOC and mitotic spindle.

**Conclusions/Significance:**

SBDS co-localizes with the mitotic spindle, suggesting a role for SBDS in the cell division process, which corresponds to the decreased proliferation capacity of SDS-patient bone marrow CD34^+^ hematopoietic progenitor cells in our culture system and also to the neutropenia in SDS patients. A role in chromosome missegregation has not been clarified, since similar spatial and time-dependent localization is observed when patient-derived SBDS mutant proteins are studied. Thus, the increased risk of myeloid malignancy in SDS remains unexplained.

## Introduction

Shwachman-Diamond Syndrome (SDS; also known as Shwachman-Diamond-Bodian Syndrome) was first described in 1964[1], [2]. The symptoms of this rare, hereditary disease include pancreatic insufficiency, skeletal abnormalities including metaphyseal dysostosis and bone marrow (BM) failure[1], [2]. The most prominent hematological symptom is neutropenia, (lowered peripheral blood neutrophil cell count), which is observed in most patients, while in 30–40% of the SDS patients also other blood cell deficiencies, like anemia (lowered red blood cell counts) and thrombocytopenia (lowered platelet counts), were observed[3]–[5]. Intriguingly, SDS patients have a highly increased risk of developing myelodysplasia and/or acute myeloid leukemia (MDS/AML) at young age[6].

In 2001, the genetic defect of SDS was mapped to the centromeric region of chromosome 7 and in 2003 the defect was narrowed down to a single gene, which was tentatively named the *Shwachman-Bodian-Diamond Syndrome (SBDS)* gene[7], [8]. In 90% of the SDS patients mutations in the *SBDS* gene were identified and this has provided the molecular basis for investigations into the underlying mechanisms defective in SDS[7]. The two most common mutations in the *SBDS* gene are 183–184TA>CT and 258+2T>C [4], [7], [9], [10], which are located in *SBDS* exon 2 and intron 2. These mutations result in a premature stop-codon (K62X) and a splice-defect that causes a frameshift resulting in a premature stopcodon (C84fsX3), respectively. It has been reported that SDS patients have residual, low levels of SBDS expression due to a very low, but correct residual splicing of the 258+2T>C SBDS transcript[11]–[13]. To date, there are no reports confirming expression of truncated SBDS proteins in SDS patients, although it can be expected that low levels of these truncated proteins are produced since SBDS mutant mRNA expression can be readily detected. Low level SBDS protein expression in SDS patients is in line with the studies with *Sbds*-deficient mice and cells, which demonstrated that SBDS is crucial for cellular viability [14] and that complete absence of SBDS expression is incompatible with life.

To date, the molecular function of SBDS remains to be further clarified, although several elegant studies have shown its involvement in RNA and/or ribosome processing and maturation[15]–[18]. Molecular analysis of other bone marrow failure syndromes, like Blackfan-Diamond anemia and dyskeratosis congenita, have revealed that in these disorders genes encoding ribosomal or related proteins are mutated. This underlines the idea that ribosomes and translational-related processes are important for proper bone marrow function[19], [20]. Most interestingly, *Nucleophosmin* (*NPM1*), a frequently mutated gene in leukemia, which encodes for a multifunctional nucleolar protein implicated in both ribosome biogenesis and centrosome duplication, was recently shown to interact with SBDS[15], [21]. This SBDS-nucleophosmin interaction would support the accepted concept of a RNA/ribosome processing defect in SDS.

At the cellular level, several lines of evidence indicate that SBDS plays a pivotal role in cell viability and/or proliferation. SBDS was shown to be widely expressed, with highest expression levels in highly proliferative cell types[7], [14]. More recently, Austin et al. have shown that SBDS is located at the mitotic spindle of primary human bone marrow stromal cells[12]. Moreover, their studies revealed that SDS patient cells (EBV B-cells and fibroblasts) are more prone to genomic instability[12].

Consistent with the observation of lowered peripheral blood neutrophil cell counts in SDS patients are studies with mouse primary granulocytic precursor cells and the 32D cell line which revealed highest SBDS expression levels in the progenitor cells[22], [23]. Knock-down of SBDS expression in 32D cells resulted in reduced cell proliferation, but normal neutrophil differentiation[23]. In contrast, in primary mouse hematopoietic progenitors, decreased SBDS expression levels resulted in impaired differentiation, but had no effect on proliferation[22]. Despite these studies in mouse neutrophilic cells and in human bone marrow (BM) stromal cells, the exact role of SBDS in human neutrophil development, remains to be established.

Here we show that SBDS expression is downregulated during neutrophil differentiation in the human myeloid leukemia cell line PLB-985 and in clinically relevant human primary CD34^+^ hematopoietic progenitor cord blood differentiation cultures. We show that SBDS co-localizes with the microtubules and centrosome of the mitotic spindle and to some extent with the microtubule organizing centre (MTOC) in interphase cells in neutrophilic cells. A direct interaction of SBDS with tubulin was shown in an *in vitro* microtubule binding assay. Most interestingly, we observed that neutrophil differentiation and proliferation in liquid cultures of SDS BM progenitors is hampered as compared to control BM hematopoietic progenitors. These data correlate with our previous studies revealing a dramatic decrease in BM granulocyte-macrophage progenitor (CFU-GM) activity in SDS patients[4] and with studies by others in mouse cells[22], [23]. Altogether, our data indicate a role for SBDS in the cell division in human myeloid progenitor cells, which may provide an explanation for the observed neutropenia and leukemia in SDS.

## Materials and Methods

### Blood cells cell culture and transfections

Peripheral blood and bone marrow aspirates were obtained after informed written consent from healthy controls or SDS patients (for additional data and mutation analysis, see Table S1). The study was approved by the Medical Ethics Committee of the Academic Medical Centre (AMC) in Amsterdam and in accordance with the Declaration of Helsinki. PBMCs and neutrophils were isolated as described[4].

Myeloid PLB-985 and HL-60 cells were cultured in RPMI with 10% FCS/penicillin (200 µg/ml)/streptomycin/4 mM L-glutamine. PLB-985 cells were differentiated with 0.5% dimethylformamide (DMF; Sigma) for 5–7 days. HeLa, U2OS and Cos-7 cells were cultured in IMDM supplemented with 10% FCS/200 µg/ml penicillin/streptomycin/4 mM L-glutamine. For transfections Fugene6 Reagent (Roche Diagnostics, Almere, The Netherlands) was used according to manufacturers' instructions.

Hematopoietic CD34^+^ progenitor cells were isolated and cultured as described[24]. Briefly, Hematopoietic CD34^+^ progenitor cells were isolated with MACS (Miltenyi Biotec, Bergisch Gladbach, Germany) from BM aspirates or Cord Blood after Ficoll PaquePLUS (GE Healthcare, Chalfont St. Giles, UK) separation yielding a CD34^+^ cell purity after MACS separation of 85–95%. Cells were cultured in IMDM with 10% FCS/penicillin (200 µg/ml)/streptomycin/4 mM L-glutamine/50 µM β-mercaptoethanol. Cells were seeded at a concentration of 0.1–0.3×10^6^ cells/ml and were supplemented with SCF, Flt-3, GM-CSF, G-CSF and IL-3 at the start of the culture. From day 7 onwards cells were supplemented only with G-CSF. Every 3 days fresh medium was added and cells were resuspended at a concentration of 0.5×10^6^ cells/ml. At several time points cell counts were determined and cytospins were made for morphological analysis. After 17 days of culture, cells were analyzed for CD11b expression by FACS with FITC-conjugated CD11b (Sanquin Reagents, Amsterdam, The Netherlands).

### Functional tests

NADPH-oxidase activity was assessed as hydrogen peroxide release determined by an Amplex Red kit (Molecular Probes Breda, The Netherlands). Cells (0.25×10^6^/mL) were stimulated with zymosan (1 mg/mL) or serum-treated zymosan (STZ; 1 mg/mL) or PMA (100 ng/mL), in the presence of Amplex Red (0.5 µM) and horseradish peroxidase (1 U/mL). Fluorescence was measured at 30-sec intervals for 30 min with the HTS7000+ plate reader. Maximal slope of H_2_O_2_ release was assessed over a 2-min interval, as described previously[4].

Intracellular calcium measurements were performed upon Fluo-3 loading (Molecular Probes, Breda, The Netherlands) for 30 minutes at 37°C and tested for intracellular calcium mobilization at a cell concentration of 2−4×10^6^ cells/ml, according to the protocol described previously for neutrophils[4].

### SBDS cloning and antibody generation

Full length SBDS was PCR-amplified from leukocyte cDNA with the following primers: SBDS forward 5′-GAGATCGGATCCTCGATCTTCACCCCCACC-3′ and reverse 5′-GAGATCGTCGACTCATTCAAATTTCTCATCTCCT-3′. The PCR product was directionally cloned with BamH1 and Sal1 into the pGEX-6P-1 vector (Amersham Pharmacia Biotech, Roosendaal, The Nederlands) to generate a GST-SBDS fusion protein and sequence verified. Recombinant GST-SBDS protein was produced and purified. The GST-tag was removed with PreScission protease and recombinant full length SBDS was used to generate a polyclonal antibody by immunization in rabbits (Eurogentec, Herstal, Belgium). For overexpression studies SBDS was directionally cloned into pEGFP-C1 (Clonetech, Leusden, The Netherlands) vector or into pCDNA3 vector (Invitrogen Breda, The Netherlands) containing a HA-tag[25].

### Immunization

Human SBDS was cloned, expressed and purified for the immunization of two different rabbits at Eurogentec according to standard procedures (Herstal, Belgium). Two booster immunizations had taken place 2–3 weeks apart. Both anti-SBDS antibody containing sera were recognizing SBDS without major differences in Western blotting and immunofluorescence staining, either used as pure sera or after protein-A purification. Whole polyclonal rabbit serum from one of the rabbits was used for the Western blotting and immunofluorescence studies shown.

### RNA isolation

RNA was isolated with Nucleospin RNA isolation system (Macherey-Nagel, Duren, Germany) according to manufacturers' instructions, as mentioned in Material and Methods. cDNA was generated with Superscript III system (Invitrogen Breda, The Netherlands) according to manufacturers' instructions.

### Western Blotting

HeLa and Cos-7 cells were lysed with 20 mM Tris-HCl pH7.4/135 mM NaCl/1.5 mM MgCl2/1%TritonX-100/10%glycerol and human leukocytes were lysed in Laemlli sample buffer. Proteins were separated with SDS-PAGE and blotted to PVDF membranes (Biorad, Veenendaal, The Nederlands). Blots were blocked with non-fat milk, incubated with primary antibodies overnight at 4°C, followed by HRP-conjugated secondary antibodies. Proteins were visualized by enhanced chemiluminescence detection (Pierce Biotechnology, Rockford, USA). Antibodies used: HA (Y11, Santa Cruz, USA), 12CA5 monoclonal (a kind gift from B.Burgering), beta-actin (AC-15; Sigma, St.Louis, USA); α-tubulin (DM1a; Sigma), SBDS polyclonal antibody (see above).

### Immunofluorescence

Cells were fixed with 4% paraformaldehyde/PBS and stained as described[25]. Secondary antibodies were Alexa488 or Alexa543-conjugated (Molecular Probes, Breda, The Netherlands) and DNA was stained with TO-PRO-3 iodide (Molecular Probes). Pictures were made with a Zeiss Axiovert 100 M confocal microscope with Zeiss 65x oil objective and processed with LSM 510 software.

### RNA expression analysis

RNA isolated with Nucleospin RNAII system (Macherey-Nagel, Duren, Germany) and cDNA was generated with SuperscriptIII reverse transcriptase (Invitrogen, Carlsbad, USA) according to manufacturers' instructions. Quantitative RT-PCR was performed as described[26] with the following SBDS primers: forward 5′-AGATAGAACGTGCTCACATGAGGC-3′, and reverse 5′-GGTGTCATTCAAATTTCTCATGTC-3′. Glucuronidase primers were used as normalization control.

### Quantative RT-PCR

Intron-spanning primers were designed to specifically amplify cDNA and exclude amplification of genomic DNA, yielding products of 100 bp for glucuronidase (GUS). The following primers were used: glucuronidase (GUS): forward primer, 5′-GAAAATATGTGGTTGGAGAGCTCATT-3′ and reverse primer, 5′-CCGAGTGAAGATCCCCTTTTTA-3′.

Amplification by PCR was performed on a LightCycler instrument (Roche, Almere, The Netherlands), with software version 3.5. The reaction was performed with LightCycler FastStart DNA MasterPLUS SYBR Green I (Roche). The annealing temperature used for all primers was 60°C. For amplification, the following LightCycler protocol was used. Taq polymerase was activated by hot-start for 10 minutes at 95°C; next the template was amplified for 40 cycles of (5 seconds at 95°C, 30 seconds at 60°C, 15 seconds at 72°C). SYBR green fluorescence was measured at the end of each cycle at 72°C. At the end of 40 cycles, melting curve analysis was performed to confirm PCR product specificty.

### Tubulin binding assay

BL21 bacteria transfected with pGEX-6P-1-SBDS were used to produce recombinant GST-SBDS protein, which was purified with Glutathione Sepharose 4 Fast Flow beads (Amersham Pharmacia Biotech). The GST-tag was removed with PreScission Protease according to the manufacturers' instruction (GE Healthcare) and SBDS protein was dialyzed against 80 mM Hepes, 1 mM EGTA, 1 mM MgCl2 and supplemented with 0.02% Triton X-100. SBDS protein was pre-cleared at 100.000 g for 20 minutes at 4°C and used in our microtubule binding protein spin-down assay according to manufacturers' instructions (Cytoskeleton, Denver, USA). Protein fractions were separated on SDS-PAGE and Coomassie stained.

### Statistics

For the comparison of mRNA concentrations as well as for hematopoietic CD34^+^ progenitor growth the analysis was performed according to the non-parametric Mann-Whitney-U test to determine statistical significance. For the distribution of cellular differentiation in BM cultures were also tested in a non-parametric manner. Cellular fractions are interdependent (totaling 100%) and were analyzed as 4-to-2 table, comparing the 4 different differentiation stages as separate categories between controls and patients (Chi-square test). For the statistical analyses we have used SPSS.15.0.1 software. In general, a *p*-value <0.05 was considered statistically significant.

## Results

### SBDS antibody generation

**Figure 1:**
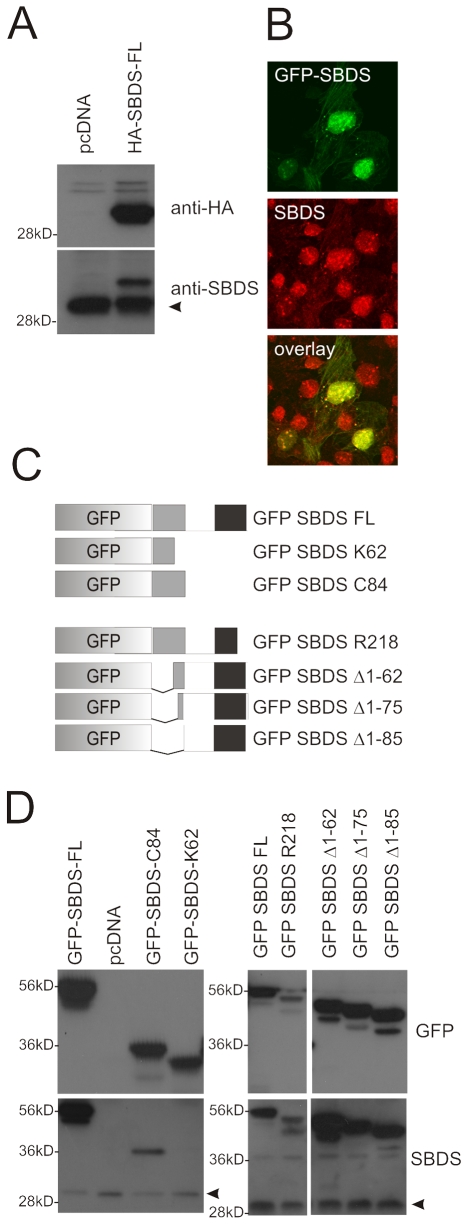
Generating SBDS Antibody. To test specificity of our SBDS antibody we overexpressed GFP- or HA-tagged SBDS in HeLa or Cos-7 cells (A) Western blotting shows that the anti-HA antibody recognizes a 30–34 kD protein in HA-SBDS-FL transfected Cos-7 cells. In a duplicate blot we show that our SBDS antibody recognizes specifically a 30–34 kD protein in HA-SBDS transfected cells. Moreover, we detected an approximately 30 kD band, which represents the endogenous SBDS. (B) Immunostaining with our SBDS antibody shows that GFP-SBDS expression (green; top picture) and endogenous stained SBDS (red; middle picture) coincides in Cos-7 cells (bottom picture). (C) Schematic overview GFP-SBDS constructs used for transient transfection followed by Western blotting analysis. The grey, white and black regions indicate the three main SBDS protein regions, namely an N-terminal FYSH domain, a central helix-turn-helix motif and the C-terminal common fold which has homology with a RNA-Recognition Motif (RRM). (D) Western blot analysis for several GFP-SBDS protein isoforms transiently expressed in HeLa cells. GFP staining (top panel) shows that these constructs are expressed at expected molecular weight and staining with our SBDS antibody shows that the antibody recognizes GFP-SBDS-FL, R219, C84 and the N-terminally truncated SBDS isoforms (GFP-SBDS Δ1–65, Δ1–75 and Δ1–85), but not GFP-SBDS-K62. Protein standard indicates protein size as indicated (56 kD, 36 kD and 28 kD, respectively)

In order to investigate SBDS protein expression, we generated a polyclonal antibody by immunizing rabbits with full length SBDS protein. Western blot analysis showed that the antibody recognizes the transiently expressed HA-tagged SBDS full length protein at the expected molecular mass of 30–34 kD (Fig. 1A) and the endogenously expressed SBDS protein of approximately 30 kD (indicated by the arrow head) in Cos-7 cells. Also immunofluorescence stainings with the SBDS antibody on GFP-SBDS-FL transfected Cos-7 cells (Fig. 1B) showed overlapping staining patterns. To further characterize the antibody, we performed Western blot analysis for several transiently expressed GFP-tagged SBDS isoforms in Hela cells (Fig. 1C). As shown in Fig. 1D, our antibody recognizes the GFP-SBDS-FL and GFP-SBDS-C84 protein, but not the GFP-SBDS-K62 protein. Furthermore, we observed that our polyclonal antibody also recognizes the C-terminal part of the SBDS protein (Fig. 1D). Thus our anti-SBDS polyclonal antibody recognizes the SBDS C-terminus and the peptide sequence between amino acids K63 and C84, but definitely not the SBDS N-terminus.

### SBDS expression is down-regulated during neutrophil differentiation

Most SDS patients have lowered numbers of peripheral blood neutrophils, suggesting that the process of neutrophil proliferation and/or differentiation is disturbed at some stage. Since mature peripheral blood neutrophils express relatively low levels of SBDS (not shown), we speculated that SBDS is playing a (more) prominent role during the early stages of neutrophil differentiation. In order to substantiate this idea, we monitored the expression of SBDS during neutrophil differentiation.

**Figure 2:**
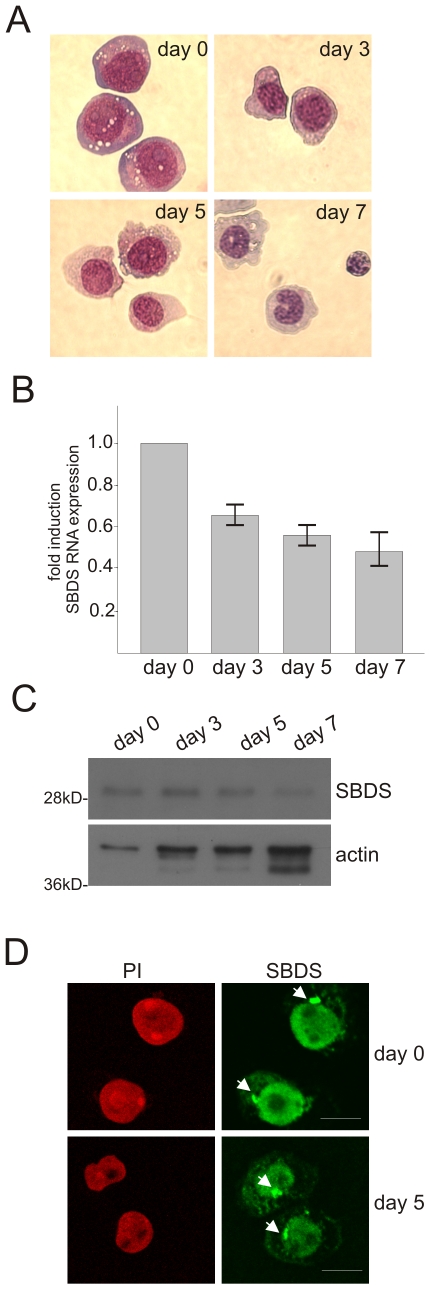
SBDS expression is downregulated during PLB-985 neutrophil differentiation. (A) PLB-985 cells were cultured for several days in the presence of 0.5% DMF to induce differentiation. May-Grünwald-Giemsa staining of cytospins of differentiating PLB-985 cells. (B) Representative quantitative RT-PCR analysis shows that SBDS mRNA expression decreases 1.8 fold with PLB-985 neutrophil differentiation (n = 5), showing the mean±SEM. (C) Western blot analysis for SBDS expressing levels and actin as a protein loading control in undifferentiated (day 0) and differentiating (day 3–7) PLB-985 cells shows that SBDS protein expression decreases during neutrophil differentiation (representative for n = 5 independent cellular differentiation experiments). (D) Immunofluorescence staining for SBDS shows that SBDS localizes prominently to the nucleus (counterstained with propidium iodide) and to lower extent in the cytoplasm. A prominent perinuclear SBDS-enriched structure (indicated with the arrow head) was detected in PLB-985 cells.

First, we examined human myeloid leukemia PLB-985 cells that were differentiated towards neutrophil-like cells based on morphological (Fig. 2A) and functional parameters, including NADPH oxidase activity and C5a receptor function (Fig.|S1). Quantitative RT-PCR analyses revealed that SBDS mRNA levels decreased during neutrophil differentiation. In the myeloid progenitors we detected 1.7–1.8 fold higher SBDS mRNA expression as compared to differentiated neutrophil-like cells (Fig. 2B). Similar data on SBDS mRNA downregulation over time were observed in the original HL-60 myeloid cell line differentiated into neutrophil-like cells (n = 3; data not shown), confirming the data in PLB-985, which represents a subclone of the HL-60 cell line.

In line with the SBDS mRNA downregulation, we also observed that the SBDS protein levels decreased during PLB-985 neutrophil differentiation (Fig. 2C). Similar to the HeLa cells, immunofluorescence staining for SBDS localization in PLB-985 cells revealed that SBDS protein is localized prominently to the nucleus with a lower extent of staining in the cytoplasm (Fig. 2D). Interestingly, distinct perinuclear SBDS enrichment in these PLB-985 cells was also observed (see also below).

**Figure 3:**
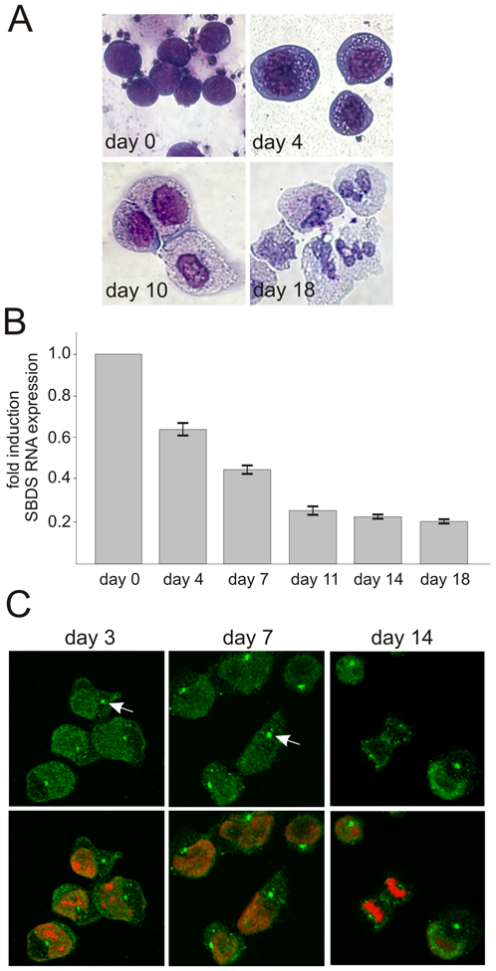
SBDS expression is downregulated during human cord blood CD34^+^ neutrophil differentiation. Human CD34^+^ cord blood cells were differentiated towards neutrophils with a cocktail of cytokines over a period of 18–21 days. (A) May-Grünwald-Giemsa staining on differentiating CD34^+^ cells shows that these cells are acquiring morphological characteristics of neutrophilic cells during the culture. (B) Representative quantitative RT-PCR analysis shows that SBDS mRNA expression decreases 5-fold with human cord blood neutrophil differentiation (n = 3; mean±SEM). (C) Immunofluorescence staining for SBDS in differentiating cord blood hematopoietic progenitor and neutrophilic cells shows that SBDS protein is localized predominantly in the nucleus and to a lower extent in the cytoplasm. In all cells a prominent SBDS-enrichment is observed in the perinuclear region (indicated with arrow). In mitotic cells, we observed that SBDS is localized at the mitotic spindle and/or centrosomes during the anaphase of mitosis (right panel).

To extend these findings, we employed a human hematopoietic progenitor CD34^+^ *in vitro* liquid neutrophil differentiation system[24] for studying SBDS expression. As revealed by morphological analysis of May-Grünwald-Giemsa stained cells, these cells differentiated over a period of 18-21 days towards neutrophilic cells (Fig. 3A). In line with the results from the PLB-985 cells, we observed a 2.5–5 fold higher SBDS RNA expression level in the CD34^+^ progenitor cells (Fig. 3B). Thus, SBDS expression is relatively high in myeloid progenitors and is down-regulated during human neutrophil differentiation in both a myeloid cell line as well as in primary hematopoietic progenitor CD34^+^ cord blood cells. To examine the SBDS subcellular localization we performed SBDS immunostainings (Fig. 3C). Highest SBDS levels were detected in the nucleus and lower levels in the cytoplasm in these hematopoietic progenitors, including the typical perinuclear localization (indicated with arrow) observed at all stages of differentiation (Fig. 3C).

### SBDS co-localizes with microtubule organizing center and the mitotic spindle

**Figure 4:**
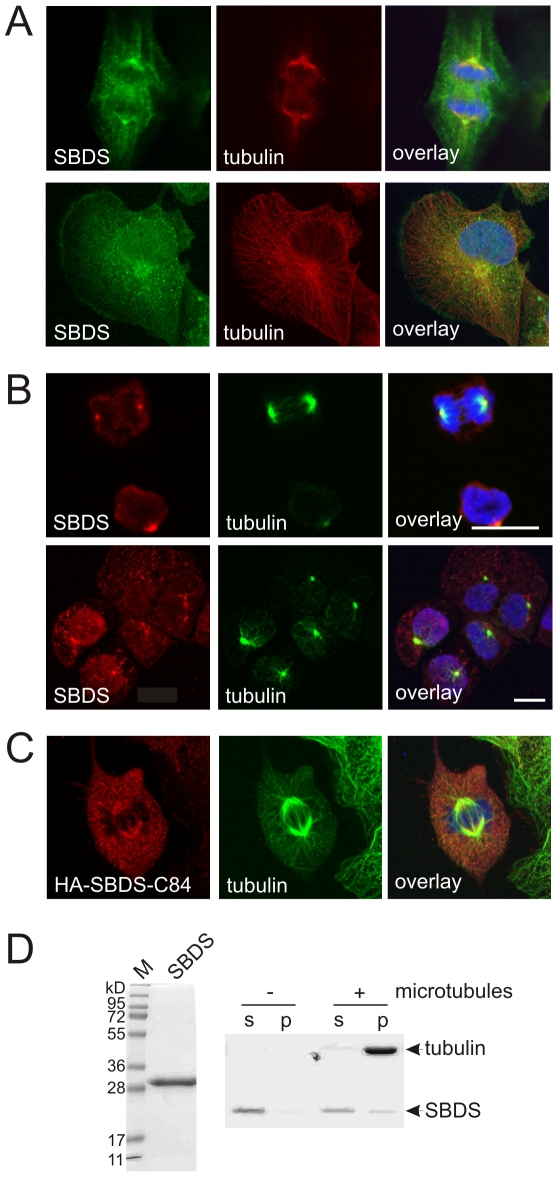
SBDS co-localizes with the MTOC and the mitotic spindle. Endogenously expressed SBDS co-localizes with the mitotic spindle and/or centrosomes (top panel) and the MTOC area (bottom panel) in (A) HeLa cells (n = 8) and in (B) cord blood CD34^+^ cells (n = 3). (C) Overexpressed HA-SBDS-C84 localizes to the mitotic spindle in U2OS cells (n = 5). (D) Recombinant expressed SBDS was used as a ligand in a microtubule-binding protein spin-down assay. Right panel shows a Coomassie-stained gel with 5 µg of the purified SBDS protein with a molecular weight of 30 kD. Left panel shows a Coomassie- stained gel with SBDS protein incubated in the presence or absence of microtubules, as indicated by tubulin staining. In the absence of microtubules, the SBDS protein remains in the soluble fraction (s) and in the presence of 0.16 nM taxol-stabilized microtubules, a small, but significant fraction of the SBDS protein co-precipitates with microtubules in the pellet (p) fraction. Representative result of 5 independent experiments.

In the above described cultures of the differentiating hematopoietic progenitor cells we noted that SBDS was located at fiber-like structures and/or the centrosomes near the segregating chromosomes (Fig. 3C). To further investigate the apparent co-localization of SBDS and the mitotic spindle, we stained the human HeLa cell line for α-tubulin and SBDS. In mitotic HeLa cells, we observed endogenously expressed SBDS at the mitotic spindle including the centrosomes (Fig. 4A, top panel). In interphase cells, SBDS was perinuclear enriched and co-localized with a central tubulin structure, also known as microtubule-organizing center (MTOC) (Fig. 4A, bottom panel). Identical results were obtained for GFP-SBDS-FL and HA-SBDS-FL overexpressed proteins in HeLa and in Cos-7 cells (not shown).

Similar to what we observed in HeLa cells, cord blood-derived CD34^+^ hematopoietic progenitor cells that were differentiated towards neutrophilic cells revealed co-localization of SBDS with the centrosomes of the mitotic spindle (Fig. 4B, top panel). The perinuclear SBDS enrichment co-localized with the MTOC in interphase hematopoietic cells (Fig. 4B, bottom panel). While in the hematopoietic progenitors the MTOC region as stained by α-tubulin appears as a distinctive, small organelle, this appears as a more diffuse region, including also organelles like the Golgi apparatus, in HeLa cells and other non-hematopoietic cell lines tested thus far.

In 90% of the SDS patients mutations in the *SBDS* gene have been observed. The two most common mutations introduce premature stopcodons due to a point mutation (K63X) and a splice defect (C84fsX3). Despite these mutations, SDS patients have been reported to have low levels of SBDS expression due correct residual splicing of the 258+2T>C SBDS transcript causing the C84fsX3 mutation [11]–[13]. Besides low level full length SBDS protein expression, it can be assumed that also these truncated proteins are expressed at (very) low levels in SDS patients.

To investigate whether these truncated SBDS proteins could potentially co-localize with the mitotic spindle and possibly disrupt proper spindle function and, as a result, the cell division process, we overexpressed GFP and HA-tagged SBDS-K62 and SBDS-C84 in HeLa cells and other cell lines. Although 5- to 10-fold lower levels of expression were obtained for these truncated SBDS proteins compared to the full-length SBDS protein (data not shown), we observed that, similar to the full-length protein, the mutant SBDS-K62 and SBDS-C84 proteins co-localized with the mitotic spindle in the cell types (Fig. 4C; and data not shown).

To further investigate the interaction of SBDS with microtubules, we purified recombinant full-length SBDS (Fig. 4D; left panel) and performed an *in vitro* microtubule protein binding assay. In the absence of microtubules, SBDS remained in the soluble fraction, while in the presence of microtubules we observed that a significant fraction of approximately 10% of the SBDS protein co-purified with the microtubules in the pellet fraction, indicating a direct interaction of SBDS with microtubules. Thus, SBDS and SBDS-patient protein isoforms co-localize with the mitotic spindle and/or centrosomes in both HeLa cells, other adherent cell types as well as in human hematopoietic cells. Also, we observed a direct interaction of the full length SBDS protein with microtubules. Together, our data suggest that SBDS is playing a role in some microtubule and/or centrosome-related function, since SBDS interacts with *in vitro* microtubules and co-localizes *in vivo* with microtubule structures in both mitotic as well as interphase cells.

### SBDS plays a role in proliferation of BM myeloid progenitors

High SBDS expression levels in myeloid progenitors, combined with the observation that SBDS co-localizes with the mitotic spindle, suggested that SBDS could play a role in the proliferation of developing myeloid progenitor cells. Previous studies have shown that SDS BM cells have decreased granulocyte-macrophage progenitor (CFU-GM) activity[4]. However, due to technical limitations these studies could not quantitatively address the proliferation rate of the progenitor cells nor analyze the different stages of neutrophil differentiation stages within these cultures. To examine in a more quantitative manner the impact of SBDS deficiency on the neutrophil differentiation and proliferation process, we employed a liquid neutrophil differentiation system. Control and SDS patient BM CD34^+^ hematopoietic progenitor cells were cultured in this *in vitro* differentiation system and cellular proliferation and differentiation was monitored over a period of 17 days.

**Figure 5:**
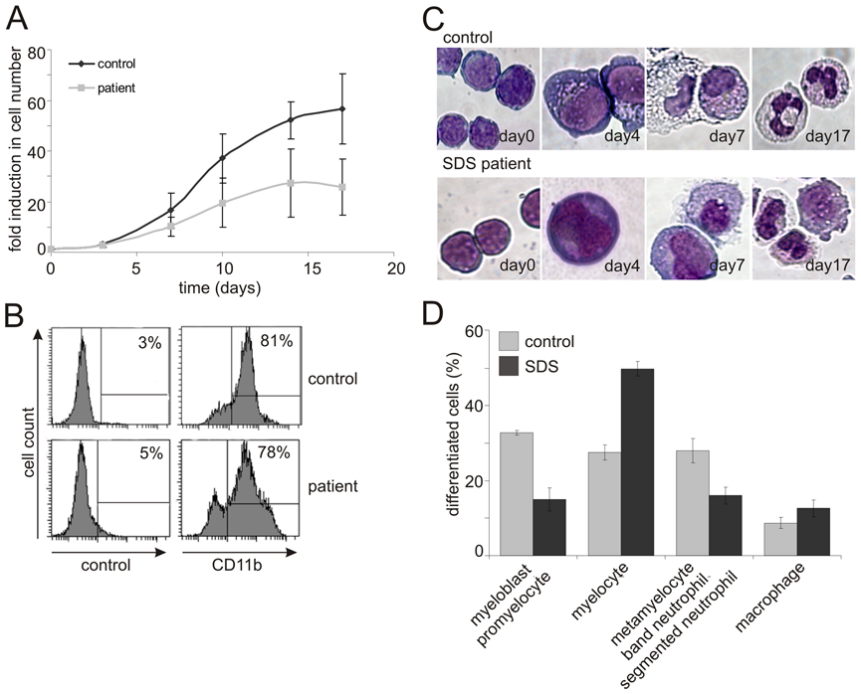
SBDS plays a role in myeloid progenitor proliferation. BM CD34^+^ cells were differentiated towards neutrophils and monitored at several stages for proliferation and differentiation. (A) Average fold induction of cell numbers from 5 independent SDS and 3 independent control BM CD34^+^ differentiation cultures. Control BM cultures show an overall significantly higher fold induction in the cell number as compared to SDS patient cells. Fold induction was calculated within each experiment by dividing the number of cells in the culture at each time point by the number of cells that was seeded at day 0. Both for controls and SDS patients 0.15−0.5×10^4^ cells were seeded. Thereafter, the average fold was calculated. Error bars indicate SEM (Statistical analysis according to Mann Witney U testing, p = 0.014). (B) FACS analysis at day 17 shows that the percentage of CD11b^+^ cells in the SDS and control cultures is approximately 80% (control n = 3; SDS patient n = 2). (C) Representative May-Grunwald-Giemsa stained cells of control or SDS patient BM cultures at various days of the culture (control n = 3; SDS patient n = 5). (D) Bars indicate the average percentage of cells of the indicated differentiation status at day 17 of the culture. SDS BM differentiation cultures (dark bars) show less terminally differentiated neutrophils as compared to the control culture (light bars). However, the percentage of intermediately differentiated (myelocytic) cells and the percentage of macrophages was (slightly) increased in SDS patient cultures (n = 3 for control and n = 4 for SDS patient. Per experiment 196–232 cells were examined. 4×2, Chi-square test, p<0.03). May-Grünwald-Giemsa stained cells were morphologically analyzed and scored by two independent persons in a blind fashion.

During the first week of SDS BM progenitor expansion we observed on average 10-fold expansion in the number of cells, while in control cultures we observed a 16.5-fold expansion in cell number on average (Fig. 5A). This difference became even more dramatic after 2 weeks of culture in which we observed a 27-fold expansion in cell number for SDS BM progenitors, whereas in the control cultures the cell number was increased 52-fold.

To examine whether the CD34^+^ hematopoietic progenitor progenitors in this differentiation culture system actually differentiated towards neutrophils, we performed flow cytometric analysis for CD11b at the end stage of the cultures (i.e. day 17). CD11b is a marker expressed by macrophages and by differentiating neutrophils from the myelocyte stage onwards[27]. In control BM cultures we observed 81% CD11b^+^ and in the SDS BM culture 78% CD11b^+^ cells, indicating that the percentage of early myeloid progenitors is similar (Fig. 5B). To analyze the neutrophil differentiation status in more detail, we stained cells with May-Grünwald Giemsa (Fig. 5C) and scored the amount of early progenitors (promyelocytes and myeloblasts), intermediately differentiated cells (metamyelocytes) and more terminally differentiated cells (metamyelocytes, band and segmented neutrophils) based on morphological characteristics on day 17 of culture (Fig. 5D). This revealed that SDS BM differentiation cultures contain a 1.9-fold reduction of terminally differentiated cells and a 1.6-fold increase of intermediately differentiated cells as compared to control cultures at day 17. Additionally, SDS cultures contain slightly increased percentages of macrophages and decreased percentages of immature progenitors (p<0.03). Thus, besides a decrease in cell growth, the SDS BM differentiation cultures also revealed a subtle but significant change in differentiation status.

## Discussion

SBDS is widely expressed in several organs and cell types. However, clinical manifestations in SDS patients are largely restricted to the pancreas and myeloid cells. Previous studies had already shown that in human SDS BM granulocyte-macrophage progenitor (CFU-GM) activity is dramatically decreased as compared to control BM cells, clearly indicating defects in the hematopoietic progenitor compartment[4], [28]. However, to date no studies have addressed the role of SBDS and the neutrophil differentiation defect in primary human cells in a more detailed fashion. The data presented here, indicate that SBDS expression levels decrease during human neutrophil differentiation, indicating that SBDS plays its most prominent role in myeloid progenitors as compared to their differentiated progeny. In line with this, we revealed that the differentiation capacity of SDS BM hematopoietic progenitor CD34^+^ progenitors is slightly impaired as compared to control progenitor cells in our *in vitro* liquid BM cultures. Additionally, the cell growth of SDS BM progenitors is significantly decreased. Our observations are consistent with the previously observed decrease in granulocyte-macrophage progenitor (CFU-GM) activity in SDS BM as compared to controls, as well as with the observed lowered peripheral blood neutrophil cell counts in SDS patients. Recently, two studies regarding the role of SBDS in mouse neutrophil differentiation were published by Yamaguchi et al. and Rawls et al., and they showed that lowering *Sbds* expression in murine hematopoietic progenitor cells or myeloid cell lines resulted in decreased myeloid cell growth and/or decreased differentiation[22], [23]. Similar to our studies in human cells, Yamaguchi et al. showed that in mouse 32D cells SDBS expression levels are down-regulated upon neutrophil differentiation[23]. Additionally, they observed that cell growth in these *Sbds* knock-down cultures was lowered as compared to control cultures. The cell growth deficit was attributed to increased apoptosis in these *Sbds* knock-down cells. Consistent with this, we also observed a slightly increased percentage of apoptotic cells at day 17 in the SDS BM cultures (6.4% of the SDS cells versus 3.0% in the control cultures; n = 3; data not shown).

Rawls et al. did not report any difference in cell growth or apoptosis in primary murine *Sbds* knock-down cells, but observed a neutrophil differentiation deficit[22] similar to our observations in the human BM hematopoietic progenitor CD34^+^ neutrophil differentiation cultures in SDS patients. In both Rawls' and our studies, intermediate differentiated cells were increased at the expense of terminally differentiated cells (i.e. segmented and band neutrophils). The similarities between our study and both of the murine myeloid cell studies confirm the requirement of SBDS during the neutrophil differentiation and proliferation process in both mice and humans and corresponds well to the clinical observation of neutropenia in SDS patients.

Our data show that human SBDS co-localizes with microtubules and centrosomes in both interphase and mitotic cells. This observation reveals a possible regulatory role for SBDS in proliferation and/or chromosome segregation. Cell division defects caused by defective SBDS function, such as incorrect chromosome distribution over the two daughter cells, can potentially result in aneuploidy and contribute to chromosome instability. This can ultimately result in an increased risk to develop malignancy, such as the observed highly increased risk of SDS patients to develop AML. Our studies are consistent with the recently published studies by Austin et al. who reported that SBDS co-localizes with the mitotic spindle in bone marrow stromal and skin fibroblast cell lines [12].

Austin et al. have reported that in SDS patient cells, a higher percentage of multipolar spindles can be detected during the cell division process and that this resulted in genomic instability, as determined by flow cytrometric DNA content analyses in these SDS patient cells[12]. This is an intriguing observation, since fibroblasts are not known to behave abnormal in SDS patients and the only malignant derangement in SDS patients leads to acute myeloid leukemia (AML). Moreover, solid tumors have thus far not been reported in SDS. In limited analyses of SDS and control BM hematopoietic and lymphoid proliferation and differentiation studies we have not observed any multipolar spindles in mitotic cells. It is unclear whether too few cells were analyzed in our studies or whether the difference in cell type, culture or analysis methods used in our and their studies contributes to this discrepancy.

In addition to our studies in primary human *SBDS* deficient cells, we have employed a model system in which we overexpressed either GFP- or HA-tagged full-length SBDS or patient-derived SBDS mutant protein isoforms (i.e. SBDS-K62 and SBDS-C84) in human cell lines in order to study the effect of these truncated SBDS proteins. We did not observe clear effects on the overall integrity of the microtubule cytoskeleton or on mitotic spindle structure (CO unpublished data). Additionally, U2OS cells transfected with GFP-SBDS-full length, GFP-SBDS-C84 or GFP-SBDS-K62 that were selected for expression and cultured over a period of 41-70 days did not reveal genomic instability (i.e. >4n DNA content), as determined by flow cytometric DNA content analysis (CO unpublished data). Nevertheless, preliminary single cell studies revealed that SBDS full length or SDS-patient derived protein isoform overexpression does result in longer cell cycle length and mitotic abnormalities at low frequency, suggesting that SBDS protein expression levels are important for correct cell division (CO unpublished data). These data indicate that overexpression of full-length and patient-derived SDS proteins does not necessarily result in genomic instability that is detectable at cell population level, but that individual cells can be affected in their cell division process. A small percentage of cells in which chromosomal missegregation results in a growth or survival advantage could potentially result in the development of leukemia, as observed in SDS patients. To further address the role of SBDS in the cell division process, more detailed studies are required in relevant human cellular model systems in which endogenous SBDS expression is reduced to levels observed in SDS patients and truncated SBDS proteins are examined for their effect on the chromosome segregation and cell division process.

According to our findings, SBDS co-localizes with the mitotic spindle and the MTOC in primary granulocytic progenitors, lymphocyte blasts and various cell lines. Moreover, we show that SBDS can interact directly with microtubules in an *in vitro* microtubule binding assay. This is an interesting finding since the MTOC is involved in many cellular processes, including cytoplasmic organization, intracellular transport, cell shape and polarity regulation and chromosome segregation. For these functions, the microtubules and many associating proteins in these complexes, require correct spatial and temporal organization to allow proper localization and dynamics of the MTOC and the microtubules for maintaining cellular viability and integrity[29], [30]. It is clear that abnormalities in the centrosomes, the core of the MTOC, might contribute to chromosome instability and tumor development[29]. Currently, the exact role of SBDS microtubule regulation/organization is unclear. It was recently shown that RNA and several RNA-binding proteins are required for mitotic spindle formation[31], and therefore the RNA-binding properties of SBDS might be involved in its microtubule-related function.

Important questions that remain to be answered relate to the role of SBDS in the regulation of microtubule assembly or dynamics, and the putative cell-type specificity of this function. In preliminary experiments, we observed an intact microtubule network in SDS-patient neutrophils (Fig.S2), which argues against a general role for SBDS in microtubule assembly. Further characterization of the SBDS domain(s) involved in the interaction with microtubules as well as the identification of additional interacting proteins is also required to understand this at the molecular level.

Taken together, there may be doubts about the exact biological effect of SBDS on spindle formation and subsequent risk of chromosome segregation defects. Nevertheless, in agreement with the findings of Austin et al.[12], we show the co-localization of SBDS with microtubules in primary hematopoietic cells and of tagged SBDS in cell lines. Also we show a direct interaction of SBDS with microtubules. Our data strongly implicate a role for SBDS in the process of chromosome segregation and/or the cell division process in neutrophils. This is especially interesting in light of the highly increased risk of SDS patients to develop AML. Furthermore, our data might have implications for unraveling the underlying mechanisms that contribute to AML in general.

## Supporting Information

Figure S1 Functional analysis of differentiated PLB-985 cells. PLB-985 cells that are undifferentiated or differentiated for 6 days with 0.5% DMF were tested for functional neutrophilic properties, such as oxidative burst capacity and the expression of functional chemo-attractant receptors. Differentiated HL-60 cells, which are similar to PLB-985 cells, have been reported to express the receptors for C5a and fMLP[32]. Hence, we tested differentiated PLB-985 cells for their ability to generate an oxidative burst and to induce a calcium-influx after stimulation with chemo-attractants. (A) Results of an NADPH oxidase assay to measure oxidative burst activity of undifferentiated and differentiated PLB-985 cells. Undifferentiated cells do not respond with an oxidative burst to zymosan, serum-treated zymosan (STZ) or PMA. Differentiated PLB-985 cells respond to STZ and PMA with an oxidative burst. (B) Differentiated PLB-985 cells were loaded with the fluorescent intracellular Calcium detecting dye, Fluo3-AM. During a time course measurement we stimulated cells with 10 nM C5a or 1 M fMLP and changes in the intracellular calcium levels were determined. Differentiated cells respond with a change in intracellular calcium levels upon exposure of C5a, but not fMLP, showing that these cells express a functional C5a receptor. Expression of the fMLP receptor is a characteristic of the last stages of differentiation. The observation that these cells do not express a functional fMLP receptor, indicates that the cells are not completely differentiated. In undifferentiated PLB-985 cells no response to either C5a or fMLP was detected (not shown).
(2.77 MB TIF)

Figure S2 Microtubule analysis in SDS-patient and control neutrophils. Peripheral Blood neutrophils from SDS patients and healthy controls were isolated and resuspended in Hepes complete medium (132mM NaCl, 20mM Hepes, 6mM KCL, 1mM MgSO4, 1.2mM K2HPO4, 1mM CaCl2, 5mM Glucose, 2.5% human albumin (Cealb; Sanquin reagents)) and seeded on fibronectin-coated coverslips (Fibronectin from Sigma) and allowed to attach to the fibronectin substrate for 30 minutes. Cells were fixed with 4% paraformaldehyde/PBS and processed for immunofluorescence staining as described in material and methods. Pictures were made with a Zeiss LSM510 microscope with Zeiss 65x oil objective and processed with LSM 510 software. Pictures are derived from 2 independent experiments.
(1.88 MB TIF)

Table S1 Clinical information of SDS patient and heathy controls. This table provides clinical information of the SDS patients and heathy controls that provided bone marrow aspirates that were used in this study.
(0.03 MB DOC)
