# Supplementary figures and images for: SBDS Expression and Localization at the Mitotic Spindle in Human Myeloid Progenitors
Source: PLoS One. 2009 Sep 17;4(9):e7084. doi: 10.1371/journal.pone.0007084 (PMC2738965; doi:10.1371/journal.pone.0007084)

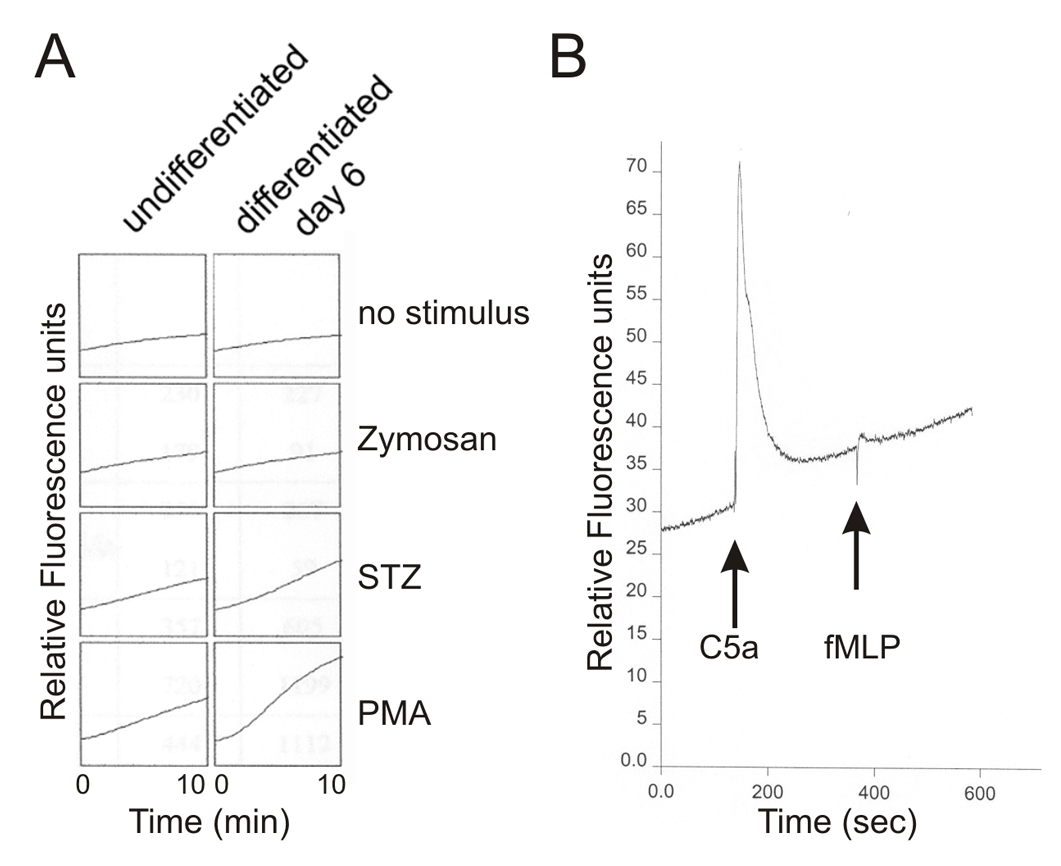

Supplement: Figure S1 — Functional analysis of differentiated PLB-985 cells. PLB-985 cells that are undifferentiated or differentiated for 6 days with 0.5% DMF were tested for functional neutrophilic properties, such as oxidative burst capacity and the expression of functional chemo-attractant receptors. Differentiated HL-60 cells, which are similar to PLB-985 cells, have been reported to express the receptors for C5a and fMLP[32]. Hence, we tested differentiated PLB-985 cells for their ability to generate an oxidative burst and to induce a calcium-influx after stimulation with chemo-attractants. (A) Results of an NADPH oxidase assay to measure oxidative burst activity of undifferentiated and differentiated PLB-985 cells. Undifferentiated cells do not respond with an oxidative burst to zymosan, serum-treated zymosan (STZ) or PMA. Differentiated PLB-985 cells respond to STZ and PMA with an oxidative burst. (B) Differentiated PLB-985 cells were loaded with the fluorescent intracellular Calcium detecting dye, Fluo3-AM. During a time course measurement we stimulated cells with 10 nM C5a or 1 M fMLP and changes in the intracellular calcium levels were determined. Differentiated cells respond with a change in intracellular calcium levels upon exposure of C5a, but not fMLP, showing that these cells express a functional C5a receptor. Expression of the fMLP receptor is a characteristic of the last stages of differentiation. The observation that these cells do not express a functional fMLP receptor, indicates that the cells are not completely differentiated. In undifferentiated PLB-985 cells no response to either C5a or fMLP was detected (not shown). (2.77 MB TIF) [file pone.0007084.s001.tif]

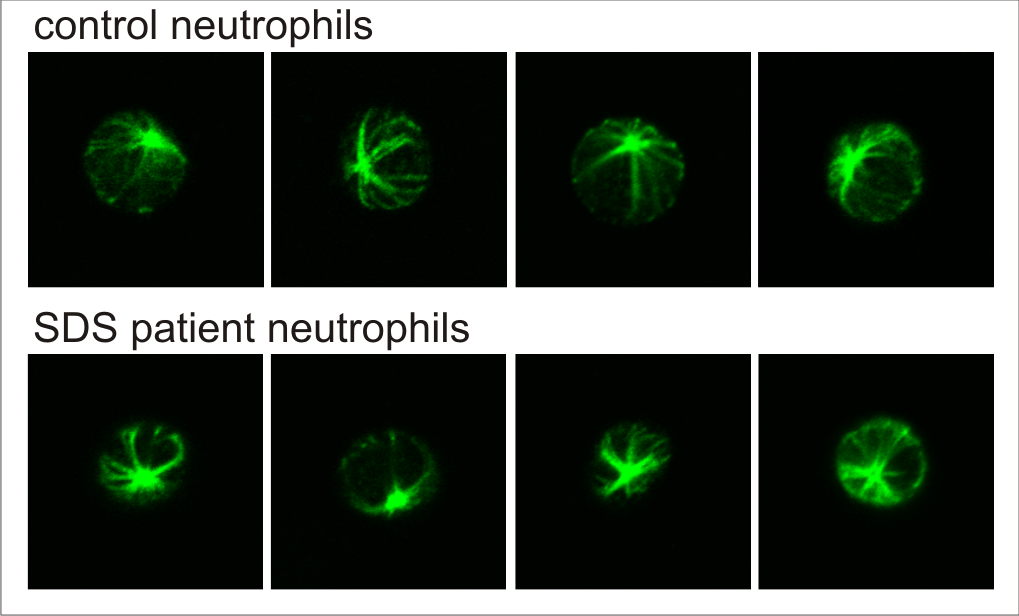

Supplement: Figure S2 — Microtubule analysis in SDS-patient and control neutrophils. Peripheral Blood neutrophils from SDS patients and healthy controls were isolated and resuspended in Hepes complete medium (132mM NaCl, 20mM Hepes, 6mM KCL, 1mM MgSO4, 1.2mM K2HPO4, 1mM CaCl2, 5mM Glucose, 2.5% human albumin (Cealb; Sanquin reagents)) and seeded on fibronectin-coated coverslips (Fibronectin from Sigma) and allowed to attach to the fibronectin substrate for 30 minutes. Cells were fixed with 4% paraformaldehyde/PBS and processed for immunofluorescence staining as described in material and methods. Pictures were made with a Zeiss LSM510 microscope with Zeiss 65x oil objective and processed with LSM 510 software. Pictures are derived from 2 independent experiments. (1.88 MB TIF) [file pone.0007084.s002.tif]
