# Supplementary material for: SBDS Expression and Localization at the Mitotic Spindle in Human Myeloid Progenitors
Source: PLoS One. 2009 Sep 17;4(9):e7084. doi: 10.1371/journal.pone.0007084 (PMC2738965; doi:10.1371/journal.pone.0007084)
Supplement: Table S1 — Clinical information of SDS patient and heathy controls. This table provides clinical information of the SDS patients and heathy controls that provided bone marrow aspirates that were used in this study. (0.03 MB DOC) [file pone.0007084.s003.doc]

**Table S1**

| **Patient* / control** | **age/gender** | **mutation** |
| --- | --- | --- |
| SDS patient 1 | 15/M | C84/E99fs |
| SDS patient 2 | 15/M | C84/K62 |
| SDS patient 3 | 22/M | C84/K62 |
| SDS patient 4 | 24/F | C84/K62 |
| SDS patient 5 | 7/F | C84/K62 |
| healthy control 1 | 18/M | - |
| healthy control 2 | 3/F | - |
| healthy control 3 | 8/F | - |

*All SDS patients suffered from moderate-to-severe neutropenia; none of the patients used Granulocyte Colony-Stimulating Factor (G-CSF). Signs or symptoms of AML/MDS in clinical, hematological BM morphology or cytogenetic tests were absent in these patients to date.
